# Homœopathy of the Present as Compared with That of Hahnemann

**Published:** 1889-12

**Authors:** M. J. Buck

**Affiliations:** Baltimore, Md.


### HOMŒOPATHY OF THE PRESENT AS COMPARED WITH THAT TAUGHT AND PRACTICED BY HAHNEMANN AND HIS IMMEDIATE FOLLOWERS.

Are we tending to fusion with allœopathy or are we drifting into allœopathy? I fear it is neither, but mongrelism. We profess Homœopathy; many of us say we understand both systems, possibly, being graduates of both schools, and we profess to select the best from both and apply to the individual cases as our judgment may direct us. Yes! and how true I am sorry to say this is in many instances, we give two, three, or four homœopathic remedies as our superior skill may dictate, alternately, of course, and leave our patient a Morphia powder to quiet his pain and cause him to sleep, and a cathartic pill to be taken in case his bowels should not move in due time, in order to get rid of some pernicious matter and accumulation of the various remedies in the alimentary track that we have given him, which of course has formed into an insoluble bolus and must be removed at all hazards; not being sure that all the indications have been met in our patient, we leave a few two-grain pills of Quinine to be taken once in two hours in case the fever should rise in our absence; and as our patient has a violent stitch in his left side which may prove obstinate, and the Acon., Bryonia, and Kali-carb. with which we have so generously supplied him fail to relieve him, we will shut up another avenue of the enemy by leaving a ℞ for Emplast-Cantharidis, 446, which we direct the nurse to paste on his side in order to draw out some materia-morbis that has accumulated between the two pleural cavities or surfaces, or possibly there may be a herd of bacteria that must be driven out through this gateway that our blister has opened; and a part of our duty would still be neglected if we were to neglect leaving a prescription for one pint of brandy, best, of course, as there is no other kind kept by druggists, hence be sure to specify best, as it always helps the nurse and attendants to know that the Doctor has an interest in his patient, and in case death should ensue the friends are doubly sure everything has been done for the poor unfortunate. Oh! how consoling it is to look up at the medicine chest after returning from the last resting place of our dear friend and see it full of heroic remedies, pills, blisters, etc.

The above enumerated are almost a fac-simile of those used in a case I recently saw which was under homœopathic care, or at least the physician was known as a homœopathic physician, a follower of Hahnemann. The mention of Hahnemann's name in conjunction with such prescribing is sufficient to cause the illustrious father to turn over in his decayed coffin and groan with pain. Yes! to have such insult offered to the memory of his masterly science, could he but rise up to-day and see how we have interpreted his teaching, he would weep tears of sorrow and regret and turn away in disgust.

Recently on a visit to one of our cities I called at a certain institution where there was a clinic being held on the eye and ear; I entered the place of healing and found a great number of patients, not less than a hundred, and five physicians in charge (presumably oculists), yet, to judge from the paraphernalia around, I might have imagined myself in a paint shop or art gallery (bar the paintings) had I not witnessed a similar condition in the allœopathic ophthalmic hospitals. Each man was armed with a brush and paint jar, or bottle of finely divided white powder looking like white lead, but evidently being lixiviated Calomel, which was being thrust in artistic style into the eyes of the poor unfortunate sufferers as they came marching by, each burying his or her face in their hands, so that when they had passed they resembled mourners at the grave in meditation, sorrow, and prayer. Of many of these patients I made inquiry of the duration of time they had been regulars of this place and the answer varied from three months to two years. I would ask you if you could imagine yourself in a homœopathic institution when witnessing such treatment? Yet such was the name it bore—a homœopathic seat of learning. Are we to be surprised at our young physicians when we know that such has been the teaching they have had? How can we expect anything different of them? Certainly not unless they act more wisely than their tutors. Is this not a deplorable state of affairs? Better by far send our students to a straight-out allœopathic institution of learning and they will not at least have their minds poisoned with bastard Homœopathy and exploded allœopathy.

In conversation with a man of eminence connected with the above-mentioned school, I asked him if he looked upon the treatment as adopted in the eye clinic as scientific Homœopathy. He admitted that it was not just Homœopathy, but that the cases were so numerous in the institution that they could not find time to treat them homœopathically. What think you of such a solution? In other words, too many to treat them properly, for such must be the literal meaning to a homœopath. I could not refrain from saying, that if they treated them according to the simillimum they would not have so many cases, *i. e.*, if they administered proper homœopathic remedies, they would succeed in curing many in one or two prescriptions, and the same patient would not remain a permanent fixture of the establishment, as is at present the fact.

You will find this class of patients going from hospital to hospital, from dispensary to dispensary, and when their patience is well nigh exhausted, they will drop into a homœopathic institution as a last resort; and can any one for a moment imagine their sorrow and disappointment when they find they are subjected to the same line of treatment, and certainly with no better results. Is it not most deplorable to waste such valuable time, and cause such unnecessary suffering, when we have in our possession such infallible means of cure if we spend but a little time in research and study of our materia medica, and have the confidence in Homœopathy we should and would have if we gave it an unbiased trial? But I fear our want of faith in what we profess, combined with mongrel teaching, is the bane of our practice. Recently I heard a sermon by an eminent divine, who, elucidating the cause of his faith (according to his manner of thinking) in God and His disciples, spoke of an example, while a theological student, of one of their number, as expressing himself as having positive convictions in his profession. Without positive conviction we are simply groping in the dark, hoping to make what we call a lucky hit; unfortunately for most of us, lucky hits are of very infrequent occurrence. Now, I urge upon all of you, unless you have positive convictions of being right, you cannot practice Homœopathy successfully. Read carefully the *Organon.* I find great pleasure in reading it. I never let a year pass by without reading it once; I find it strengthens me in the true medicine. Don't have your patient believe that you understand both systems of medicine, and that you select only that which you think is best for the individual case, for when you admit this you acknowledge your want of confidence in your own profession. He that professes all things and systems is surely a failure; a jack of all trades is master of none.

Can it be possible that *contrari contrariis curantur* is better adapted to adult members of the family, and *similia similibus curantur* is better suited to the children; if so, when do children cease to be children, and require allœopathic treatment, and *vice versa?*

If *similia similibus curantur* be the proper curative principle, then it always remains so; if it be false, it never can be true, and should be abandoned at once.

Who ever knew of two opposites both being true? Who ever knew of a successful politician professing both Republicanism and Democracy? Who ever knew of an eminent divine professing two forms of religion? They are simply incompatible, and will not mix a bit more than oil and water. So it is with Homœopathy and allœopathy. What a pleasure it is to believe you are right, and to practice according to your convictions, but a man who practices both, practices neither and has no convictions; he is simply practicing hap-hazard, and nature performs all his cures in spite of his ignorance, if any are cured.

Permit me to urge upon you the importance of simplifying your practice; don't alternate your remedies, and then when a cure is made, you know what remedy has cured the case, and it assists you in fixing your symptomatology; besides, when you get into the habit of giving two remedies, you often see the necessity of a third, and it will not require you long to see where a fourth can be interpolated with advantage. In this way your vocabulary will grow indefinitely, and you will continually be in doubt of the proper remedy. I dare say there is not one of you who would countenance a person taking four or six remedies, yet it is quite easy to imagine a case where it may be necessary if we only accustom our imagination to be elastic. Who dare question the propriety of giving four or six remedies when he admits the necessity of two? M. J. Buck, M. D.

Baltimore, Md.

